# Supplementary material for: TDP-43 upregulates lipid metabolism modulator ABHD2 to suppress apoptosis in hepatocellular carcinoma
Source: Commun Biol. 2022 Aug 13;5:816. doi: 10.1038/s42003-022-03788-w (PMC9376094; doi:10.1038/s42003-022-03788-w)

Supplementary Information (SI) for:

TDP-43 upregulates lipid metabolism modulator ABHD2 to suppress apoptosis in hepatocellular carcinoma

Supplementary Materials and methods

Cell Culture, transfection and treatments

Human liver cancer cell lines (SMMC-7721, HepG2, MHCC97L, MHCC97H and Huh-7) and HEK 293T cell lines were purchased from American Type Culture Collection (ATCC, USA). Under the conditions of 5% CO_2 and 37°C, the cells were cultured in high-glucose Dulbecco's Modified Eagle's Medium (DMEM, BI, Israel) supplemented with 10% fetal bovine serum (FBS, Gibco, USA). The indicated plasmids or siRNA were delivered into selected cells by mean of lipofectamine 2000 reagent transfection (Invitrogen, USA) according to the manufacturer's instructions. To stably knockdown TDP-43 in HCC cells, MHCC97H cells were infected with lentivirus carrying pLVX-shTDP-43-puro or pLVX-shRNA-scramble-puro plasmid; then, the cells were treated with puromycin to obtain the stable cell line with TDP-43 silencing (MHCC97H-KD-TDP-43) or control cell line (MHCC97H-Control). The sequences of the two shRNAs against TDP-43 are listed in supplementary table. The sh-TDP-43#2 was finally used in the subsequent experiments. To analyze the stability of RNA, the indicated cells were treated with actinomycin D (Santa Cruz, USA) at 2 μg/ml for the corresponding time points. In Supplementary Figure 5, the indicated cells were treated with NAC (Beyotime, China) at 5mM for 48h. In Supplementary Figure 6, the indicated cells were treated with FFA (oleic acid, sigma) at 30μM for 48h.

qRT-PCR assay

The total RNA was extracted from HCC cells using Trizol (Takara, Japan) and then converted to cDNA according to the manuals of the EasyTaq DNA Polymerase (TRNASGen Biotech, China). Using SYBR Green Master Mix (Thermo Fisher Scientific, USA) reagent, qRT-PCR was completed through Applied Biosystems quantum 3 real time PCR system (Thermo Fisher Scientific). Using the expression level of *GAPDH* gene as a reference, the expression level of

each gene was analyzed by the ΔΔCt method. The primers used in this study were synthesized by Gene Create (Wuhan, China), and the primer sequences were shown in Supplementary table 2.

Reactive oxygen species (ROS) detection

To measure the changes in the intracellular ROS levels, an oxidation-sensitive fluorescent probe DCFH-DA (Solarbio, China) was used. Briefly, the cells were laid in 6-well plates and transfected with indicated siRNAs or plasmids. After 48 h, 10 μM DCFH-DA diluted by serum-free DMEM was added and incubated for 20 min, where DMSO was used as a blank control. The Rosup provided by the kit was used as a positive control. Subsequently, the cells were washed with serum-free medium for three times, and observed under the fluorescent microscope to detect the fluorescent signal. Image J software was used to quantify the fluorescence signal.

Determination of Triglyceride（TG）content

In this experiment, intracellular total TG levels were measured by the Triglyceride Assay Kit (BC0625, Solarbio Science & Technology, China). Treated cells were collected and assayed for intracellular total TG levels according to the manufacturer's protocol. The experiment was performed separately for the blank group (A), the standard group (B), and the treated group (C). The TG content in cells was calculated according to the formula: TG (mg/10^4 cell) = (C-A)/(B-A)/cell density.

Colony formation assay

The cells were plated into 24-well plates and cultured with DMEM containing 10% FBS for 12 hours. Then, the cells were transfected with indicated plasmids or siRNAs. After 48 hours, the cells were re-suspended and plated in 6-well plate in DMEM without FBS for 20 days. The medium was changed every three days. After colony formation, it was washed twice with precooled PBS and fixed with 4% paraformaldehyde for 30 minutes. Then, the colony was dyed with 1% crystal violet at room temperature for 15 minutes and washed with PBS for three times. After natural air drying, the colony was observed and the formation efficiency was analyzed by software image J.

Dual luciferase reporter assay

Dual luciferase reporter analysis was performed according to the manufacturer's instructions (Promega, USA). The *ABHD2* 3'UTR reporter constructs were transfected into HEK 293T cells along with indicated siRNAs or plasmids. The Renilla luciferase (pRL-TK) plasmid was co-transfected into cells as an internal control. After 48 hours, the cells were lysed and the luciferase activity was detected with the dual luciferase reporter assay system (Promega).

Cell Counting Kit-8 (CCK-8)

The changes in cell proliferation were determined by Cell Counting Kit-8 (CCK-8) (Kemix, Australia). Firstly, the cells were seeded in 6-well plates, and transfected with indicated plasmids or siRNAs. After 48h, the transfected cells were digested with trypsin, and then re-laid into 96-well plates at the density of 1000 cells per well. Then, at the indicated time points, CCK-8 reagent (10μl) was added to each well and cultured for 2 hours. Finally, the OD450 was measured using a microplate reader. Six replicates were set in each group, the experiments were repeated at least three times.

Supplementary tables

Supplementary table 1

**The sequences of siRNAs and shRNAs**	
si-TDP-43#1	GCTTCGCTACAGGAATCCA
si-TDP-43#2	GGAGAGGACTTGATCATTA
si-ABHD2#1	CCTCAAGAATGCAGGCTCA
si-ABHD2#2	CAAGAATGCAGGCTCAACA
sh-TDP-43#1	GCTCTAATTCTGGTGCAGCAA
sh-TDP-43#2	GCTTTGGCTCAAGCATGGATT

Supplementary table 2

**The sequences of qRT-PCR primers**	
Bcl-2-Forward Primer	GGTGGGGTCATGTGTGTGG
Bcl-2-Reverse Primer	CGGTTCAGGTACTCAGTCATCC
p53-Forward Primer	GGAAATTTGCGTGTGGAGTATTT
p53-Reverse Primer	GTTGTAGTGGATGGTGGTACAG
TDP-43-Forward Primer	TCATCCCCAAGCCATTCAGG
TDP-43-Reverse Primer	TGCTTAGGTTCGGCATTGGA
ABHD2 -Forward Primer	GCCCCACCTGACCTCTACT
ABHD2 -Reverse Primer	AACGAAAGTGCGGATGTATT
Bax -Forward Primer	TGAAGACAGGGGCCTTTTTG
Bax -Reverse Primer	AATTCGCCGGAGACACTCG
GAPDH -Forward Primer	AATCCCATCACCATCTTCCA
GAPDH -Reverse Primer	TTCACACCCATGACGAACAT
HAL-Forward Primer	AGAATAAGCCCGACAATGGTG
HAL-Reverse Primer	TTCCACGAACTCGTTGTTCTC
ALPI-Forward Primer	TACACGTCCATCCTGTACGG
ALPI-Reverse Primer	CTCGCTCTCATTCACGTCTGG
GCH1-Forward Primer	GTGAGCATCACTTGGTTCCAT
GCH1-Reverse Primer	GTAAGGCGCTCCTGAACTTGT
PIP5KL1-Forward Primer	GCATCTCCGAGAGGTATGACA
PIP5KL1-Reverse Primer	GAGGCTGTAATCCAGCACGTT
LCLAT1-Forward Primer	CACCCTACCTGTGGCATTATTG
LCLAT1-Reverse Primer	CCATTCTTGTCCGATGGTTCAT
KLHL8-Forward Primer	GACATGGCGGATCAGTATGCC
KLHL8-Reverse Primer	GCTGCGGTGATACACTTACAA
CBARP-Forward Primer	GCCCCTCAAGATTGTCACCAT
CBARP-Reverse Primer	CTGATCTCTGCGAAGTCAGTG
LIPG-Forward Primer	GATGCGGTCAATAATACCAGGG
LIPG-Reverse Primer	CCGATCAAGTGGACATTCCCG
HPD-Forward Primer	GAAACACGGTGACGGAGTGAA
HPD-Reverse Primer	CTCCCGCATGATTTTGGCG

Supplementary table 3

Primers for the construction of ABHD2 3'UTR	
WT-Forward Primer	GTCGAAAATGCAAACTTGGG
WT-Reverse Primer	CATGCAGAGTGCTATTTCTT
Del-1-Forward Primer	GTCGAAAATGCAAACTTGGGGAGGGCAGAAAGATCAAGGCT
Del-1-Reverse Primer	CATGCAGAGTGCTATTTCTT
Del-2-Forward Primer	GTCGAAAATGCAAACTTGGG
Site-delete-reverse Primer	TGACCATTTTTTTACAGCGT
Site-delete-forward Primer	ACGCTGTAAAAAAATGGTCA
Del-2-Reverse Primer	CATGCAGAGTGCTATTTCTT

Supplementary table 4

Clinical characteristics of 32 pairs of clinical liver cancer tissues in microarray					
**No.**	**Age**	**Gender**	**Type**	**Differentiation**	**Diagnosis**
1	55	M	Hepatocellular	Low	Carcinoma
2	55	M	Hepatocellular	Low	Para-carcinoma
3	52	F	Cholangiocarcinoma	Low	Carcinoma
4	52	F	Cholangiocarcinoma	Low	Para-carcinoma
5	73	F	Hepatocellular	High	Carcinoma
6	73	F	Hepatocellular	High	Para-carcinoma
7	58	M	Nodular	-	Carcinoma
8	58	M	Nodular	-	Para-carcinoma
9	57	M	Hepatocellular	Middle	Carcinoma
10	57	M	Hepatocellular	Middle	Para-carcinoma
11	58	F	Cholangiocarcinoma	Low	Carcinoma
12	58	F	Cholangiocarcinoma	Low	Para-carcinoma
13	52	F	Cholangiocarcinoma	Low	Carcinoma
14	52	F	Cholangiocarcinoma	Low	Para-carcinoma
15	50	M	Hepatocellular	Middle	Carcinoma
16	50	M	Hepatocellular	Middle	Para-carcinoma
17	39	M	Hepatocellular	High	Carcinoma
18	39	M	Hepatocellular	High	Para-carcinoma
19	65	M	Hepatocellular	High	Carcinoma
20	65	M	Hepatocellular	High	Para-carcinoma

21	35	M	Hepatocellular	Middle	Carcinoma
22	35	M	Hepatocellular	Middle	Para-carcinoma
23	59	M	Hepatocellular	Middle	Carcinoma
24	59	M	Hepatocellular	Middle	Para-carcinoma
25	35	M	Hepatocellular	Middle	Carcinoma
26	35	M	Hepatocellular	Middle	Para-carcinoma
27	54	F	Hepatocellular	High	Carcinoma
28	54	F	Hepatocellular	High	Para-carcinoma
29	52	M	Hepatocellular	Low	Carcinoma
30	52	M	Hepatocellular	Low	Para-carcinoma
31	56	M	Hepatocellular	Middle	Carcinoma
32	56	M	Hepatocellular	Middle	Para-carcinoma
33	63	M	Hepatocellular	Middle	Carcinoma
34	63	M	Hepatocellular	Middle	Para-carcinoma
35	37	M	Hepatocellular	Middle	Carcinoma
36	37	M	Hepatocellular	Middle	Para-carcinoma
37	64	M	Mixed	Low	Carcinoma
38	64	M	Mixed	Low	Para-carcinoma
39	54	M	Hepatocellular	Middle	Carcinoma
40	54	M	Hepatocellular	Middle	Para-carcinoma
41	46	M	Hepatocellular	Middle	Carcinoma
42	46	M	Hepatocellular	Middle	Para-carcinoma
43	57	M	Hepatocellular	Middle	Carcinoma
44	57	M	Hepatocellular	Middle	Para-carcinoma
45	36	M	Hepatocellular	High	Carcinoma
46	36	M	Hepatocellular	High	Para-carcinoma
47	61	F	Mixed	Low	Carcinoma
48	61	F	Mixed	Low	Para-carcinoma
49	48	M	Hepatocellular	High	Carcinoma
50	48	M	Hepatocellular	High	Para-carcinoma
51	37	M	Hepatocellular	Middle	Carcinoma
52	37	M	Hepatocellular	Middle	Para-carcinoma
53	49	M	Hepatocellular	Middle	Carcinoma
54	49	M	Hepatocellular	Middle	Para-carcinoma
55	42	M	Hepatocellular	Middle	Carcinoma

56	42	M	Hepatocellular	Middle	Para-carcinoma
57	63	F	Hepatocellular	High	Carcinoma
58	63	F	Hepatocellular	High	Para-carcinoma
59	45	M	Hepatocellular	Middle	Carcinoma
60	45	M	Hepatocellular	Middle	Para-carcinoma
61	54	M	Hepatocellular	Middle	Carcinoma
62	54	M	Hepatocellular	Middle	Para-carcinoma
63	60	M	Hepatocellular	Low	Carcinoma
64	60	M	Hepatocellular	Low	Para-carcinoma

Note： M: Male； F: Female

Supplementary Figure 1. TDP-43 inhibits apoptosis and promotes proliferation in HCC cells.

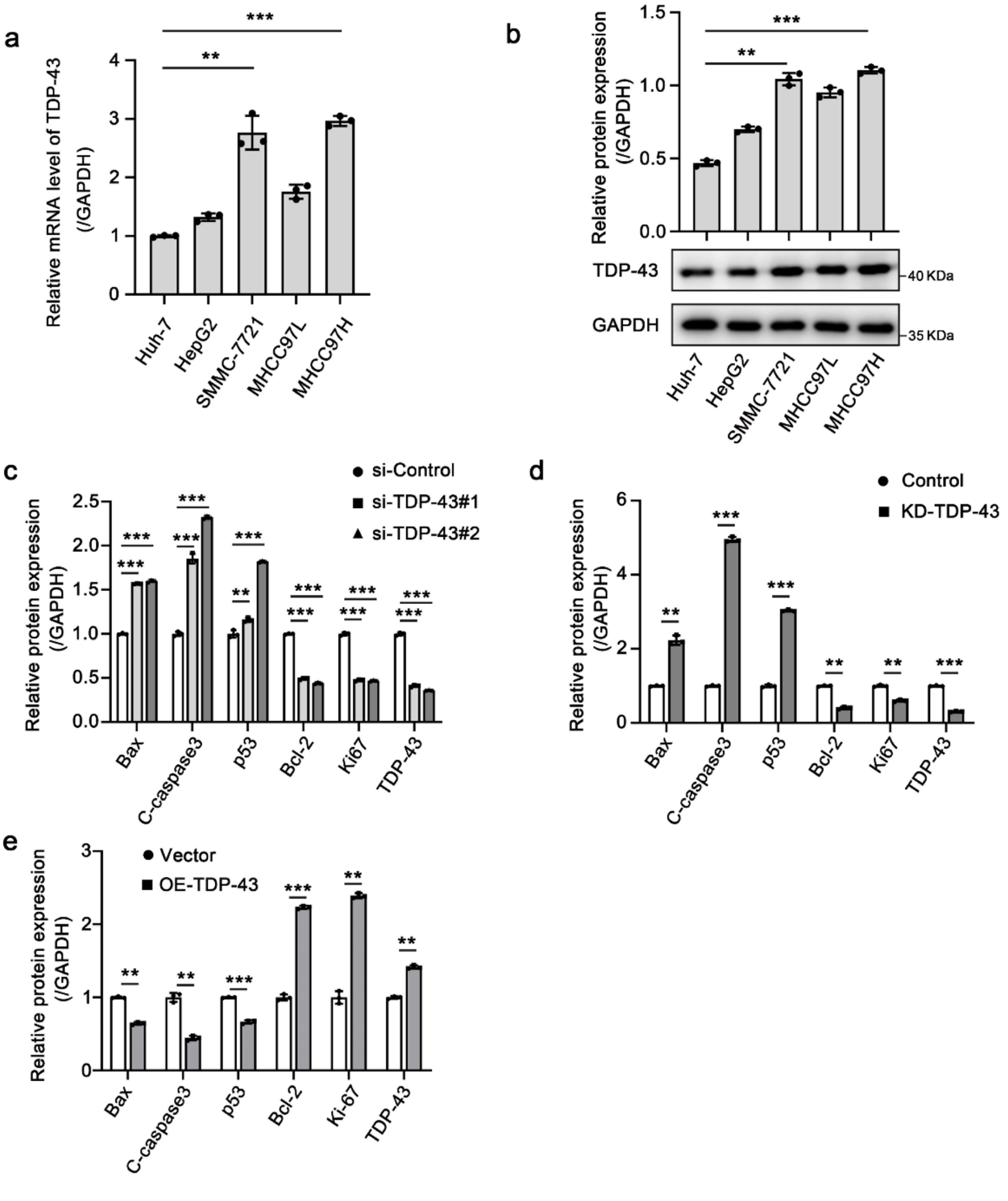

a, b qRT-PCR (a) and Western blot (b) analyses of TDP-43 in Huh-7, HepG2, SMMC-7721, MHCC97L, and MHCC97H cell lines. The upper panel in (b) shows the quantification of the intensity relative to GAPDH. **c-e** The quantification of the intensity relative to GAPDH in Fig.1i. (c), (d) and (e) separately shows the quantification data in SMMC-7721, MHCC97H and Huh-7 cell lines. The values in the graphs represent the mean of three biologically independent experiments. Error bars represent ±s. d. *P < 0.05, **P < 0.01, ***P < 0.001 by two-tailed Student's t-test.

Supplementary Figure 2. Relative number statistics of differentially expressed genes in HCC cells after knockdown of TDP-43.

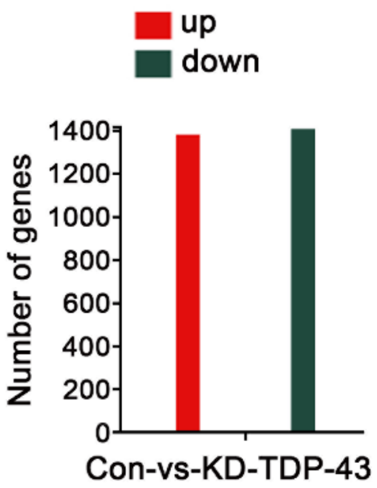

Supplementary Figure 3. TDP-43 is positively related to ABHD2 and increases ABHD2 expression in HCC.

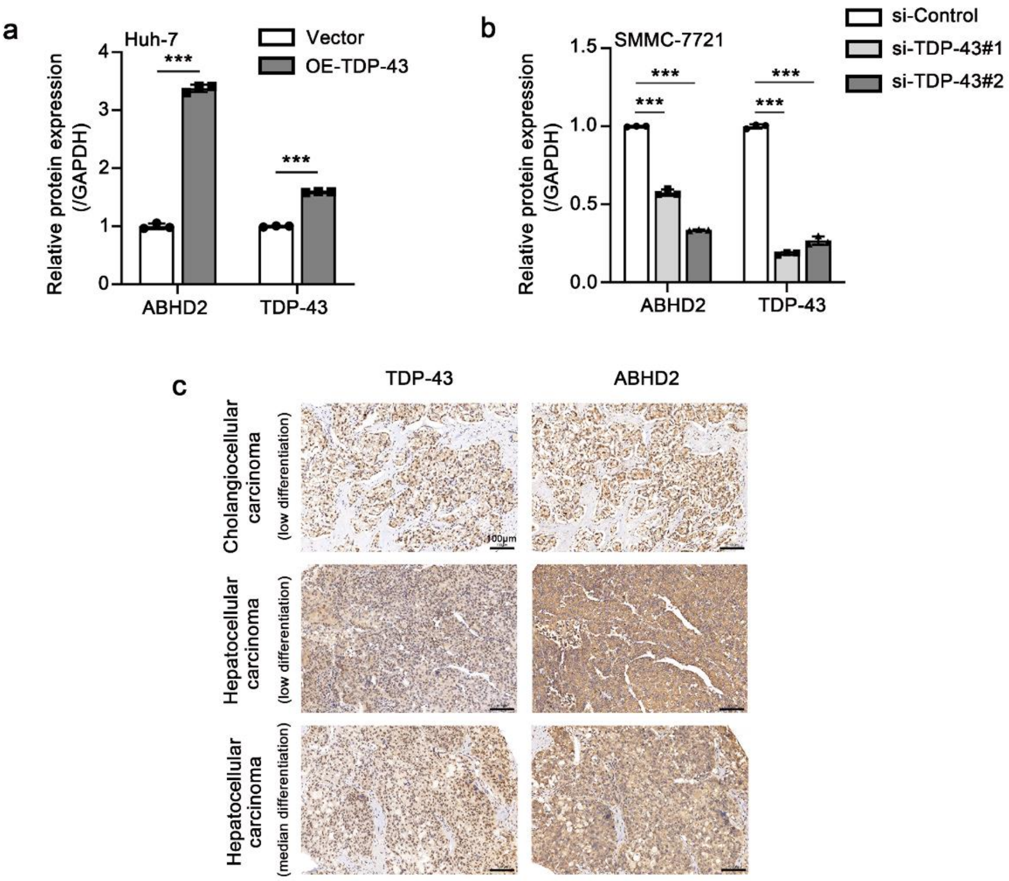

a Quantification of TDP-43 and ABHD2 protein intensity relative to GAPDH in Fig.4d (left panel). **b** Quantification of TDP-43 and ABHD2 protein intensity relative to GAPDH in Fig.4d (right panel). **c** IHC staining analysis of TDP-43 and ABHD2 expression in HCC tissue microarrays. Scale bar: 100 μm. The values in the graphs represent the mean of three biologically independent experiments. Error bars represent ±s. d. *P < 0.05, **P < 0.01, ***P < 0.001 by two-tailed Student's t-test.

Supplementary Figure 4. Lipid metabolism modulator ABHD2 contributes to the malignant behaviors of HCC.

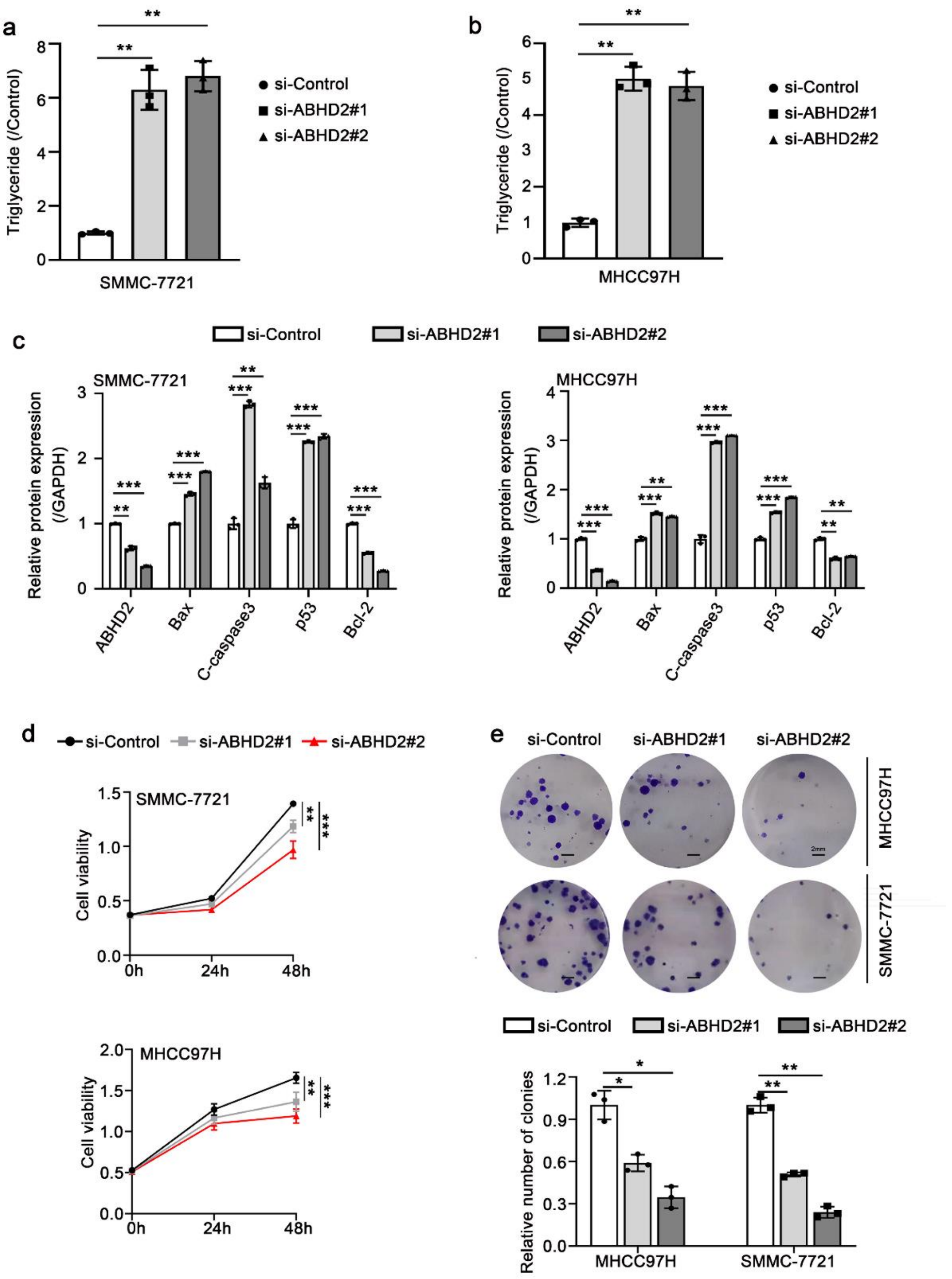

a, b Analysis of triglyceride (TG) levels in SMMC-7721 (a) and MHCC97H (b) cells. The cells were transfected with the indicated siRNAs. **c** Quantification of protein intensity relative to GAPDH in SMMC-7721 (left panel) and MHCC-97H (right panel) cell lines in Fig.6g. **d** CCK-8 assay for cell proliferation ability of SMMC-7721 (upper panel) and MHCC97H (lower panel)

cells at the indicated time points. The cells were transiently transfected with si-Control or si-ABHD2s. **e** Colony photographs (upper panel) and colony formation efficiency (lower panel) in MHCC97H and SMMC-7721 cells upon ABHD2 deletion. Scale bar: 2 mm. The values in the graphs represent the mean of three biologically independent experiments. Error bars represent ±s. d. *P < 0.05, **P < 0.01, ***P < 0.001 by two-tailed Student's t-test.

Supplementary Figure 5. NAC treatment could alleviate the inhibition of apoptosis induced by ABHD2 overexpression.

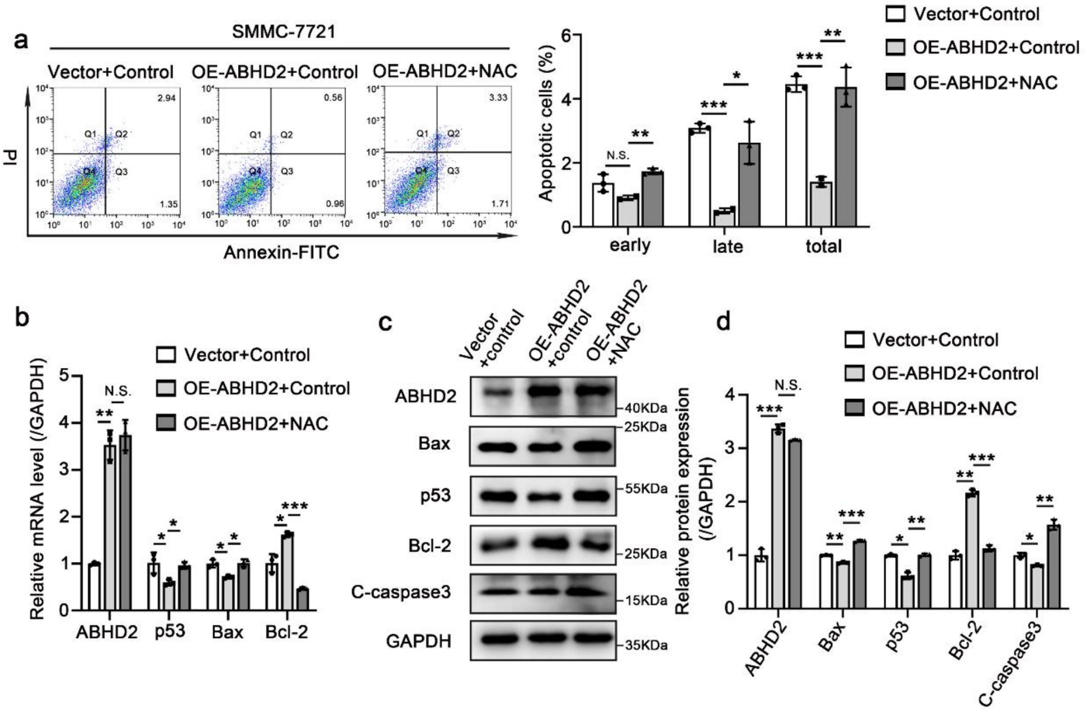

a Flow cytometry analysis of apoptosis in SMMC-7721 cells after transient transfection with the displayed plasmids upon DMSO or NAC treatment (5mM, 48h). The right panel is the statistical graphs. **b-d** qRT-PCR (b) and Western blot analysis (c) of the expression level of apoptosis markers in SMMC-7721 cells after transient transfection with the displayed plasmids upon DMSO or NAC treatment (5mM, 48h). The protein intensity relative to GAPDH was quantitatively analyzed in (d). The values in the graphs represent the mean of three biologically independent experiments. Error bars represent ±s. d. *P < 0.05, **P < 0.01, ***P < 0.001 by two-tailed Student's t-test.

Supplementary Figure 6. TDP-43 facilitates lipid metabolism *via* ABHD2 to suppress apoptosis *in vitro*.

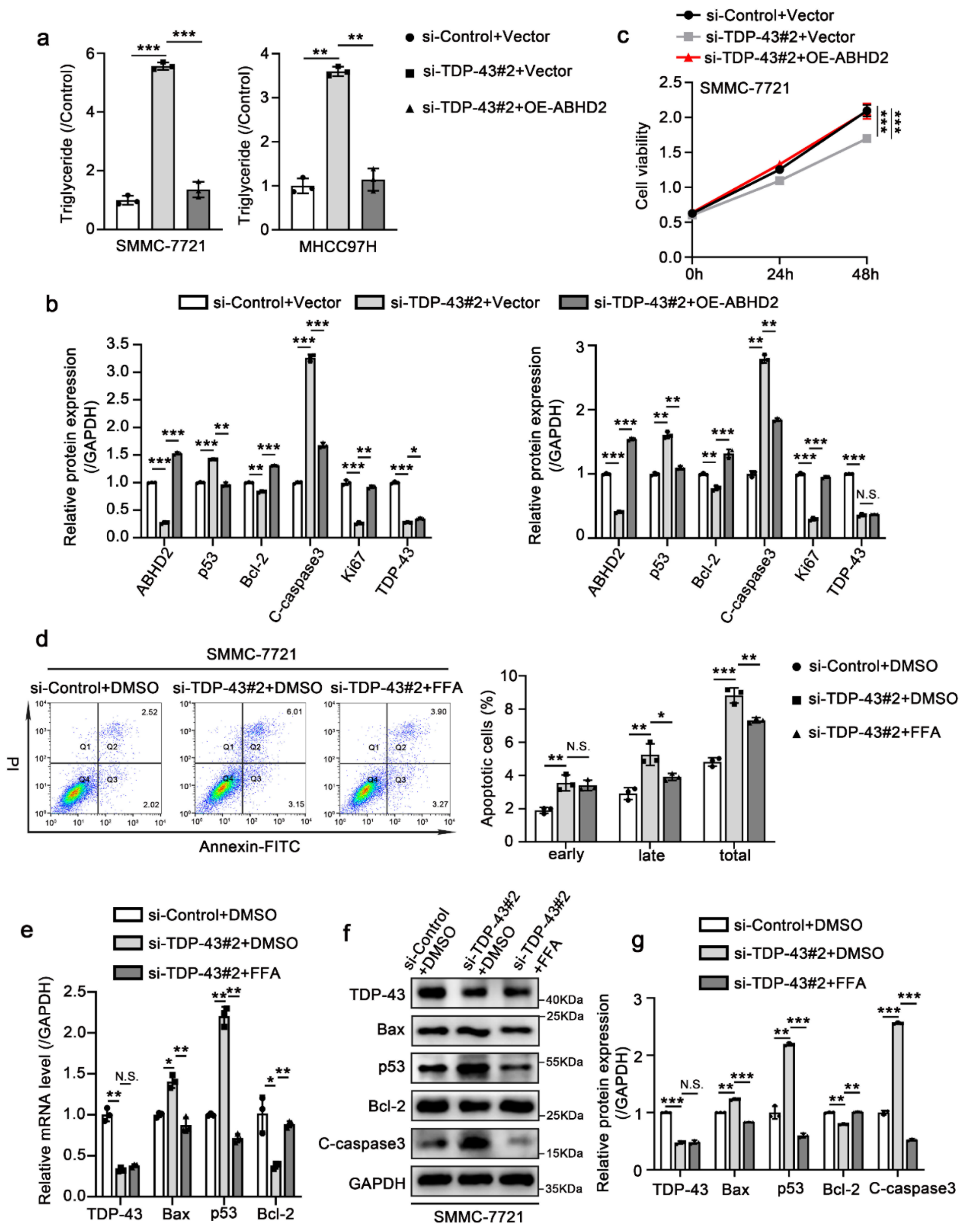

a Analysis of TG levels in SMMC-7721 (left panel) and MHCC97H (right panel) cells. The cells were transfected with the indicated siRNAs or plasmids. **b** Quantification of protein intensity relative to GAPDH in SMMC-7721 (left panel) and MHCC-97H (right panel) cell lines in Fig.7g. **c** CCK-8 assay for cell proliferation ability of SMMC-7721 cells at the indicated

time points. The cells were transfected with the indicated siRNAs or plasmids. **d** Flow cytometry analysis of apoptosis in SMMC-7721 cells after transient transfection with the displayed siRNAs upon DMSO or FFA treatment. The right panel is the statistical graphs. **e-g** qRT-PCR and Western blot analysis of the expression level of apoptosis markers in SMMC-7721 cells after transient transfection with the displayed siRNAs upon DMSO or FFA treatment. (g) shows the quantification of protein intensity relative to GAPDH. The values in the graphs represent the mean of three biologically independent experiments. Error bars represent ±s. d. *P < 0.05, **P < 0.01, ***P < 0.001 by two-tailed Student's t-test.

Supplementary Figure 7. Uncropped blots of figure 1 and supplementary figure 1.

FIG.1i

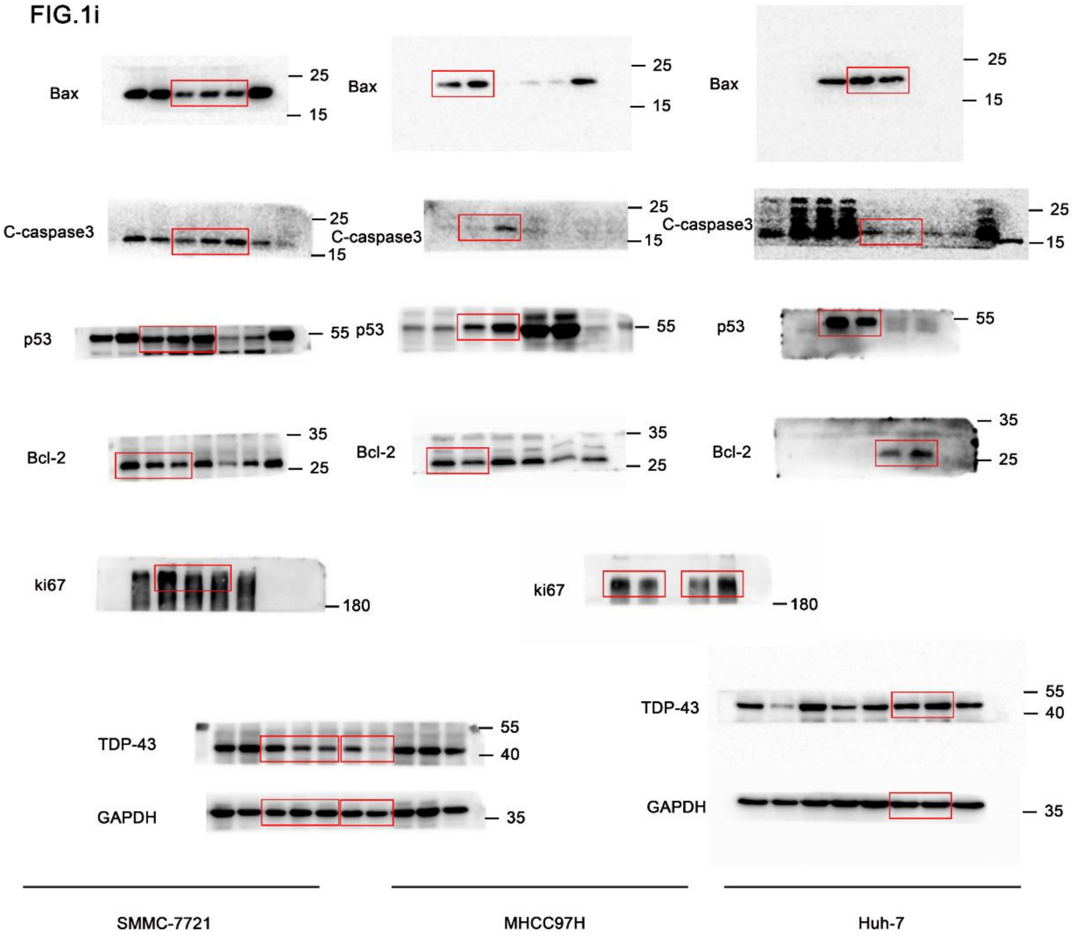

FIG.S1b

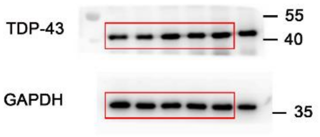

Supplementary Figure 8. Uncropped blots of figure 4 and 6.

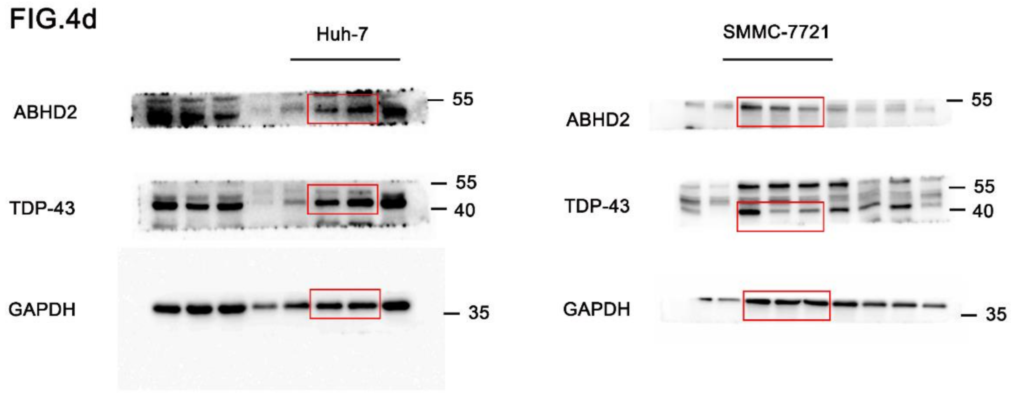

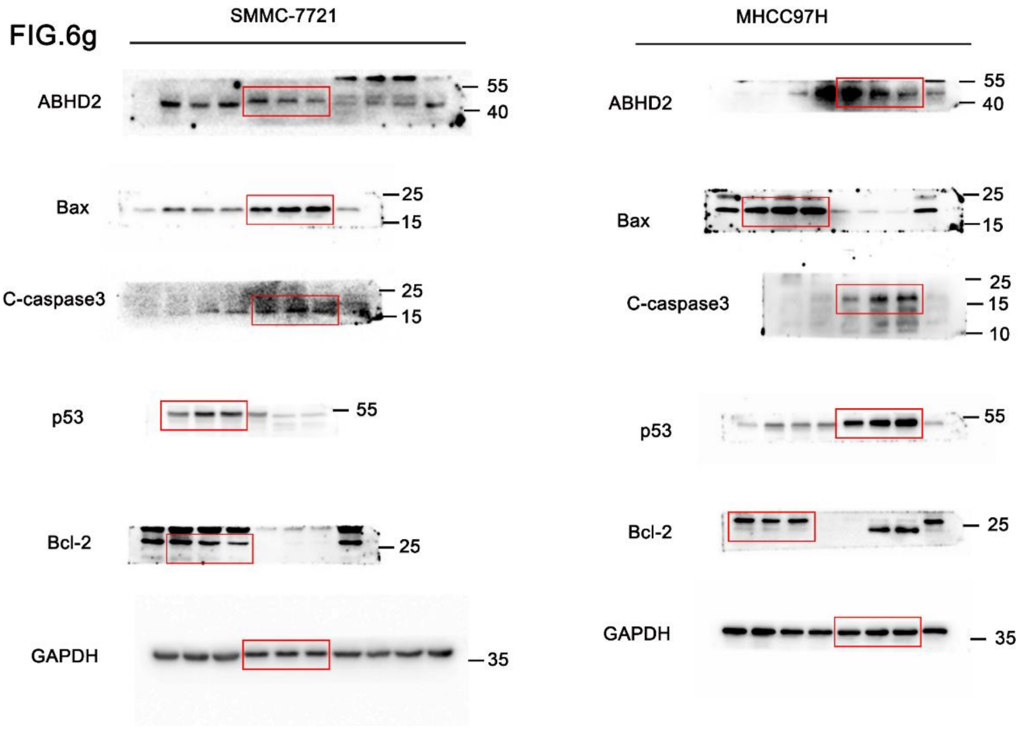

Supplementary Figure 9. Uncropped blots of Supplementary figure 5 and 6.

FIG.S5c

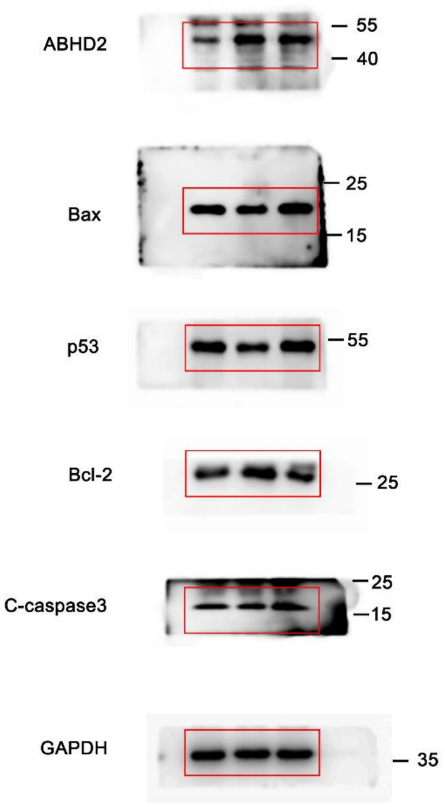

FIG.S6f

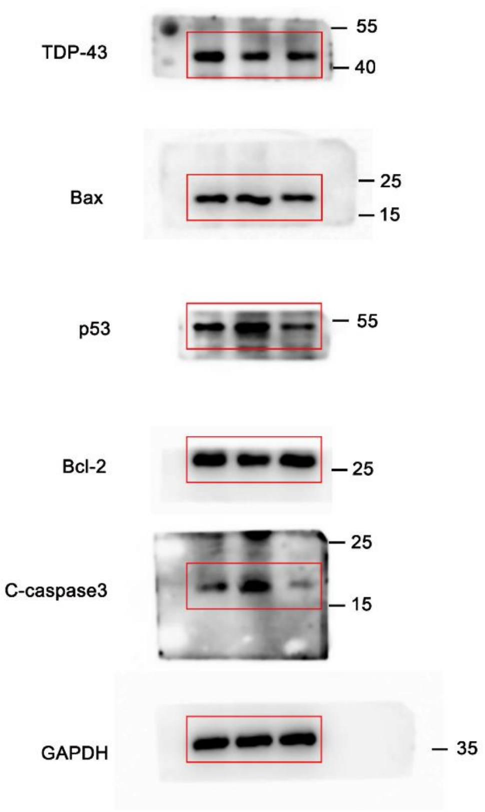

Supplementary Figure 10. Uncropped blots of figure 7.

FIG.7g

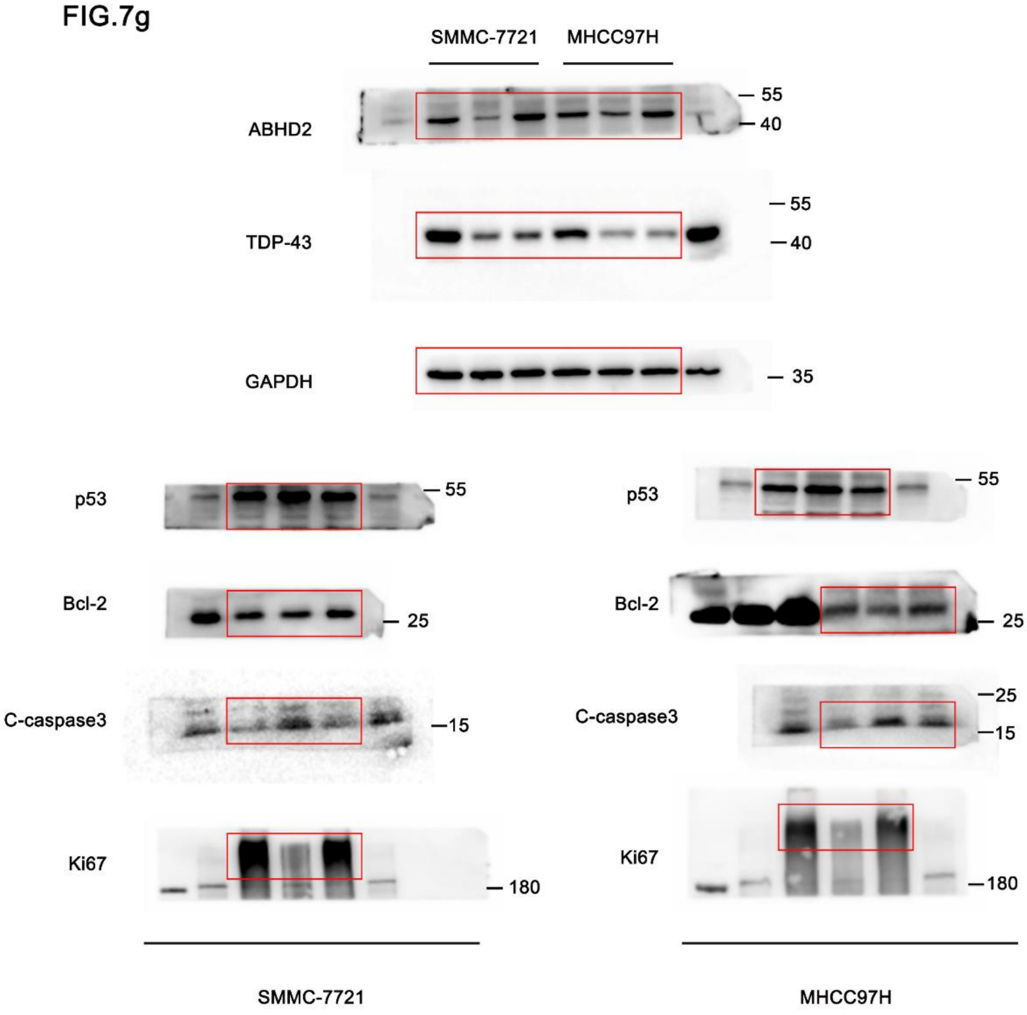

Supplement: Supplementary file 2 — Supplementary Information [file 42003_2022_3788_MOESM2_ESM.pdf]
