# Supplementary material for: TDP-43 upregulates lipid metabolism modulator ABHD2 to suppress apoptosis in hepatocellular carcinoma
Source: Commun Biol. 2022 Aug 13;5:816. doi: 10.1038/s42003-022-03788-w (PMC9376094; doi:10.1038/s42003-022-03788-w)
Supplement: Supplementary file 5 — Reporting Summary [file 42003_2022_3788_MOESM5_ESM.pdf]

nature portfolio

Corresponding author(s): Bowen Liu, Zhifa Shen

Last updated by author(s): Jul 13, 2022

Reporting Summary

Nature Portfolio wishes to improve the reproducibility of the work that we publish. This form provides structure for consistency and transparency in reporting. For further information on Nature Portfolio policies, see our Editorial Policies and the Editorial Policy Checklist.

Statistics

For all statistical analyses, confirm that the following items are present in the figure legend, table legend, main text, or Methods section.

n/a	Confirmed	
☐	☒	The exact sample size (*n*) for each experimental group/condition, given as a discrete number and unit of measurement
☐	☒	A statement on whether measurements were taken from distinct samples or whether the same sample was measured repeatedly
☐	☒	The statistical test(s) used AND whether they are one- or two-sided<br>*Only common tests should be described solely by name; describe more complex techniques in the Methods section.*
☒	☐	A description of all covariates tested
☒	☐	A description of any assumptions or corrections, such as tests of normality and adjustment for multiple comparisons
☐	☒	A full description of the statistical parameters including central tendency (e.g. means) or other basic estimates (e.g. regression coefficient) AND variation (e.g. standard deviation) or associated estimates of uncertainty (e.g. confidence intervals)
☒	☐	For null hypothesis testing, the test statistic (e.g. *F*, *t*, *r*) with confidence intervals, effect sizes, degrees of freedom and *P* value noted<br>*Give P values as exact values whenever suitable.*
☒	☐	For Bayesian analysis, information on the choice of priors and Markov chain Monte Carlo settings
☒	☐	For hierarchical and complex designs, identification of the appropriate level for tests and full reporting of outcomes
☒	☐	Estimates of effect sizes (e.g. Cohen's *d*, Pearson's *r*), indicating how they were calculated

Our web collection on statistics for biologists contains articles on many of the points above.

Software and code

Policy information about availability of computer code

Data collection	no software was used
Data analysis	Statistical significance was assessed using GraphPad Prism8. The Immunohistochemical staining quantification was analyzed by Image J.

For manuscripts utilizing custom algorithms or software that are central to the research but not yet described in published literature, software must be made available to editors and reviewers. We strongly encourage code deposition in a community repository (e.g. GitHub). See the Nature Portfolio guidelines for submitting code & software for further information.

Data

Policy information about availability of data

All manuscripts must include a data availability statement. This statement should provide the following information, where applicable:

- Accession codes, unique identifiers, or web links for publicly available datasets
- A description of any restrictions on data availability
- For clinical datasets or third party data, please ensure that the statement adheres to our policy

We declare that the data supporting the findings of this study are available within the paper and its supplementary information files.

Field-specific reporting

Please select the one below that is the best fit for your research. If you are not sure, read the appropriate sections before making your selection.

☒ Life sciences ☐ Behavioural & social sciences ☐ Ecological, evolutionary & environmental sciences

For a reference copy of the document with all sections, see nature.com/documents/nr-reporting-summary-flat.pdf

Life sciences study design

All studies must disclose on these points even when the disclosure is negative.

Sample size	No sample size was predetermined. Three or more independent samples were used to obtain results which were then used to perform statistical analysis. All sample sizes and the number of independent replicates are stated in figure legends.
Data exclusions	None.
Replication	The number of independent biological replicates are described in figure legends.
Randomization	The samples were randomly allocated into experimental group.
Blinding	The investigators were blinded to group allocation during data collection and analysis.

Reporting for specific materials, systems and methods

We require information from authors about some types of materials, experimental systems and methods used in many studies. Here, indicate whether each material, system or method listed is relevant to your study. If you are not sure if a list item applies to your research, read the appropriate section before selecting a response.

Materials & experimental systems

n/a	Involved in the study	
☐	☒	Antibodies
☐	☒	Eukaryotic cell lines
☒	☐	Palaeontology and archaeology
☐	☒	Animals and other organisms
☒	☐	Human research participants
☒	☐	Clinical data
☒	☐	Dual use research of concern

Methods

n/a	Involved in the study	
☒	☐	ChIP-seq
☐	☒	Flow cytometry
☒	☐	MRI-based neuroimaging

Antibodies

Antibodies used	GAPDH, Abcam, ab181602; TDP-43, Abcam, ab190963; ABHD2, absin, abs140798; C-caspase3, Cell signaling, #9664; p53, proteintech,10442-1-AP; Bax, Cell signaling,#5023; Bcl-2, abcam, ab182858 ;Ki67 ,Abcam, ab16667 ; Ki67 ,Abcam, ab15580
Validation	All antibodies that are commercially available have been tested for species reactivity and application by the manufacturers.

Eukaryotic cell lines

Policy information about cell lines

Cell line source(s)	The cell lines were purchased from American Type Culture Collection (ATCC).
Authentication	The authentication of all cell lines used was performed by short tandem repeat (STR)-profiling.
Mycoplasma contamination	All cell lines tested negative for mycoplasma contamination.
Commonly misidentified lines (See ICLAC register)	No misidentified cell lines were used in this study.

Animals and other organisms

Policy information about studies involving animals; ARRIVE guidelines recommended for reporting animal research

Laboratory animals	BALB/C-nu nude mice, male, 6-8 week
Wild animals	N/A
Field-collected samples	The study did not involve samples collected from the field
Ethics oversight	All animal experiments were approved by the Management Committee of Xinxiang Medical University for Medical Laboratory Animal Sciences

Note that full information on the approval of the study protocol must also be provided in the manuscript.

Flow Cytometry

Plots

Confirm that:

☒ The axis labels state the marker and fluorochrome used (e.g. CD4-FITC).

☒ The axis scales are clearly visible. Include numbers along axes only for bottom left plot of group (a 'group' is an analysis of identical markers).

☒ All plots are contour plots with outliers or pseudocolor plots.

☒ A numerical value for number of cells or percentage (with statistics) is provided.

Methodology

Sample preparation	The SMMC-7721, MHCC97H or Huh-7 cells were cultured with DMEM containing 10% FBS in 6-well plates, and then transfected with the corresponding siRNA or plasmid for 48h. After transfection, the cells were washed with precooled PBS and resuspended by binding buffer. Subsequently, the cells were dyed with 5μl Annexin V-FITC and 5μl propidium. Within 1h, the apoptotic cells were detected by flow cytometry. Three replicates were set in each group.
Instrument	we used BD FACSCaliburTM Flow Cytometer (REF 342975) to collect the data.
Software	The softwares Flow Jo and GraphPad Prism was used to collect and analyze data.
Cell population abundance	The abundance of the relevant cell populations was showed in the associated figures and figure legends.
Gating strategy	We prepared a blank group without any treatment as a negative control, and two single staining groups which only stained by FITC or PI. Then, the positive cell population was defined according to the negative cell population.

☒ Tick this box to confirm that a figure exemplifying the gating strategy is provided in the Supplementary Information.
